# Right-to-left shunt and subclinical ischemic brain lesions in Chinese migraineurs: a multicentre MRI study

**DOI:** 10.1186/s12883-018-1022-7

**Published:** 2018-02-14

**Authors:** Xiao-han Jiang, Si-bo Wang, Qian Tian, Chi Zhong, Guan-ling Zhang, Ya-jie Li, Pan Lin, Yong You, Rong Guo, Ying-hua Cui, Ying-qi Xing

**Affiliations:** 1 Neuroscience Centre, Department of Neurology, The First Hospital of Jilin University, Changchun, China; 2 Department of Neurology, People’s Hospital of Linyi City, Linyi, China; 3 Department of Neurology, Weifang People’s Hospital, Weifang, China; 4 Department of Ultrasound, The Centre Hospital of Changsha City, Changsha, China; 5 Diagnostic Ultrasound Centre, The Centre Hospital of Jilin City, Jilin, China; 6 Department of Neurology, The Second Hospital of Longyan City, Longyan, China; 7 Department of Neurology, The First Hospital of University of South China, Hengyang, China; 8 Department of Neurology Function, The People’s Hospital of Liaoning Province, Shenyang, China; 9 Centre of Head and Neck Vascular Ultrasound, Department of Neurology, The Hospital of Yanbian University, Yanji, China

**Keywords:** Infarction, Magnetic resonance imaging, Migraine, Patent foramen ovale, Transcranial Doppler ultrasonography, White matter

## Abstract

**Background:**

Migraine is considered as a risk factor for subclinical brain ischemic lesions, and right-to-left shunt (RLS) is more common among migraineurs. This cross-sectional study assessed the association of RLS with the increased prevalence of subclinical ischemic brain lesions in migraineurs.

**Methods:**

We enrolled 334 migraineurs from a multicentre study from June 2015 to August 2016. Participants were all evaluated using contrast-enhanced transcranial Doppler, magnetic resonance imaging (MRI), and completed a questionnaire covering demographics, the main risk factors of vascular disease, and migraine status. RLS was classified into four grades (Grade 0 = Negative; Grade I = 1 ≤ microbubbles (MBs) ≤ 10; Grade II = MBs > 10 and no curtain; Grade III = curtain). Silent brain ischemic infarctions (SBI) and white matter hyperintensities (WMHs) were evaluated on MRI.

**Results:**

We found no significant differences between migraineurs with RLS and migraineurs without RLS in subclinical ischemic brain lesions.SBI and WMHs did not increase with the size of the RLS(p for trend for SBI = 0.066, p for trend for WMHs = 0.543). Furthermore, curtain RLS in migraineurs was a risk factor for the presence of SBI (*p* = 0.032, OR = 3.47; 95%CI: 1.12−10.76). There was no association between RLS and the presence of WMHs.

**Conclusion:**

Overall, RLS is not associated with increased SBI or WMHs in migraineurs. However, when RLS is present as a curtain pattern, it is likely to be a risk factor for SBIs in migraineurs.

**Trial registration:**

No. NCT02425696; registered on April 21, 2015.

**Electronic supplementary material:**

The online version of this article (10.1186/s12883-018-1022-7) contains supplementary material, which is available to authorized users.

## Background

Migraine, which is the most common type of primary headache encountered in the clinic, affects daily life and even causes ischemic events in sufferers. The relationship between migraine and subclinical brain ischemic lesions, including silent brain infarctions (SBI) and white matter hyperintensities (WMHs) on magnetic resonance imaging (MRI), is complicated and disputed [1–5]. Several studies have reported that SBI and WMHs were also more prevalent in subjects with migraine [1, 3, 6], especially migraine with aura (MA) [2, 7].

Furthermore, right-to-left shunt (RLS) may be a shared risk factor in migraine and subclinical brain ischemic lesions. The relationship between RLS and migraine has been widely investigated [8–10]. Several case−control analyses have indicated that RLS is more common in patients who suffer MA than normals [10, 11]. RLS, and particularly large RLS, caused mainly by a patent foramen ovale (PFO) [12], is also considered to be a cause of cryptogenic stroke (CS) in young patients [13].

Nevertheless, the role of RLS in migraineurs with subclinical brain ischemic lesions remains uncertain. To investigate whether RLS is an etiological factor for subclinical brain ischemic lesions in migraineurs, we cooperated with eight other centres to collect subjects for uniform assessment of subclinical ischemic brain lesions in migraineurs with and without RLS by MRI. In the present study, we evaluated whether, in migraineurs, (1) RLS per se, or a particular size/subtype of RLS, is associated with a higher incidence of SBI and WMHs; (2) RLS is associated with increased SBI located in the posterior circulation; (3) deep WMHs (dWMHs) is more commonly present in subjects with RLS than those without RLS.

## Methods

### Study population

The study procedures were approved by the Ethics Committee of the First Hospital of Jilin University (clinical trial no. NCT02425696; registered on April 21, 2015).All patients provided written informed consent prior to participation. All methods were carried out in accordance with the approved guidelines.

From June 2015 to August 2016, we consecutively enrolled patients from nine medical centres in China; the patients were aged between 18 and 70 years, and were diagnosed with migraine through a questionnaire based on the International Classification of Headache Disorders, 3rd edition beta version (ICHD-3beta; Headache Classification Committee of the International Headache Society, 2013). All subjects have underwent neurological examination and were screened by transcranial Doppler (TCD), contrast-enhanced TCD (c-TCD), MRI, and a questionnaire to obtain information on demographic characteristics, the main risk factors of vascular disease, and migraine status. The main risk factors of vascular disease include body mass index (BMI), hypertension, diabetes, heart disease (including atrial fibrillation and coronary heart disease), and smoking status.

Patients with the following characteristics were excluded: (1) intracranial or extracranial artery stenosis or occlusion; (2) incomplete MRI or c-TCD, an insufficient temporal window, or who could not perform the Valsalva manoeuvre (VM) due to cognitive disorder, or severe heart or lung disease.

### c-TCD protocol

c-TCD examinations were performed by using a hand-held 2-MHz probe connected to the TCD detector (EMS-9A or 9 PB, Delica, China). The procedure was performed by experienced ultrasound technologists blinded to migraine diagnosis and MRI findings. Before the test, patients were asked to practice a standardized VM. Briefly, an 18-gauge catheter was inserted into the patient’s right antecubital vein. Contrast agent was prepared, using 9 ml isotonic saline solution, 1 ml of air, and a drop of the patient’s blood, which was mixed vigorously between two 10-ml syringes through a three-way stopcock, and was injected with the participant in the supine position [14]. After 30 mixing cycles, the contrast agent was injected as a rapid bolus while insonating the left middle cerebral artery (MCA) through the temporal bone window. The insonation lasted 20 s from the injection. The procedure was carried out three times: in the first measurement, injection was performed during normal respiration to detect any permanent RLS. The second and third injections were performed 5 s prior to the start of a 10-s VM. The time interval between injections was at least 5 min.

**Fig. 1:**
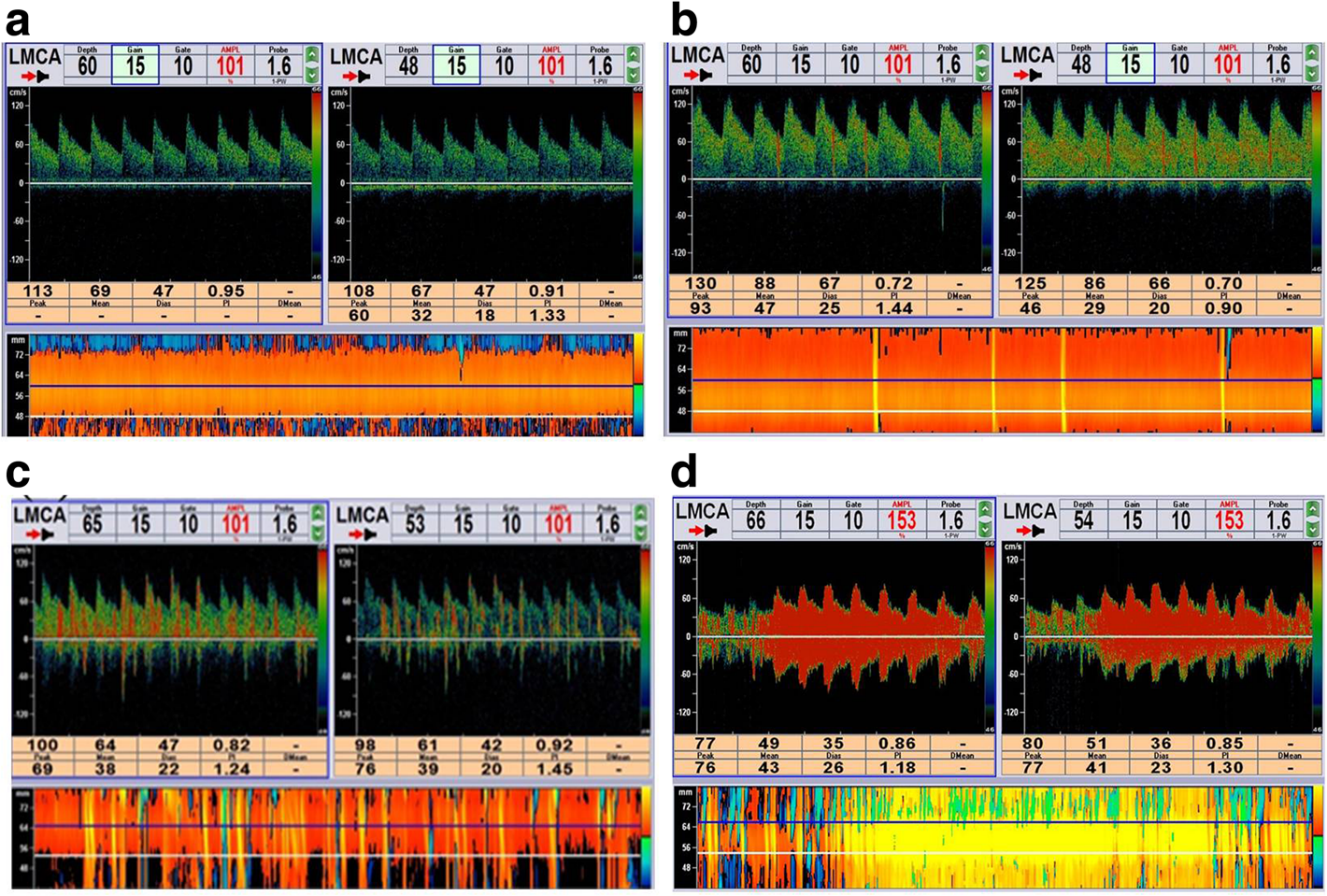
The four-level RLS categorization based on the microbubbles count. **a** Grade 0 = Negative; **b** Grade I = 1 ≤ MBs ≤ 10; **c** Grade II = MBs > 10 and no curtain; **d** Grade III = curtain. Abbreviations: RLS (right-to-left shunt); MBs (microbubbles)

The maximum number of microbubbles (MBs) was taken as the estimate of the maximum degree of shunt, which was recorded separately from the MCA during rest and after the VM [15]. On the basis of the standards reported by Serena et al., Jauss et al., and Yang et al. [15–17], a four-level RLS categorization, based on the MB count, was applied as follows: Grade 0 = Negative; Grade I = 1 ≤ MBs ≤ 10; Grade II = MBs > 10 and no curtain; Grade III = curtain (Fig. 1). RLS was considered permanent if it occurred during rest, and latent if it only occurred after a VM.

### MRI

**Fig. 2:**
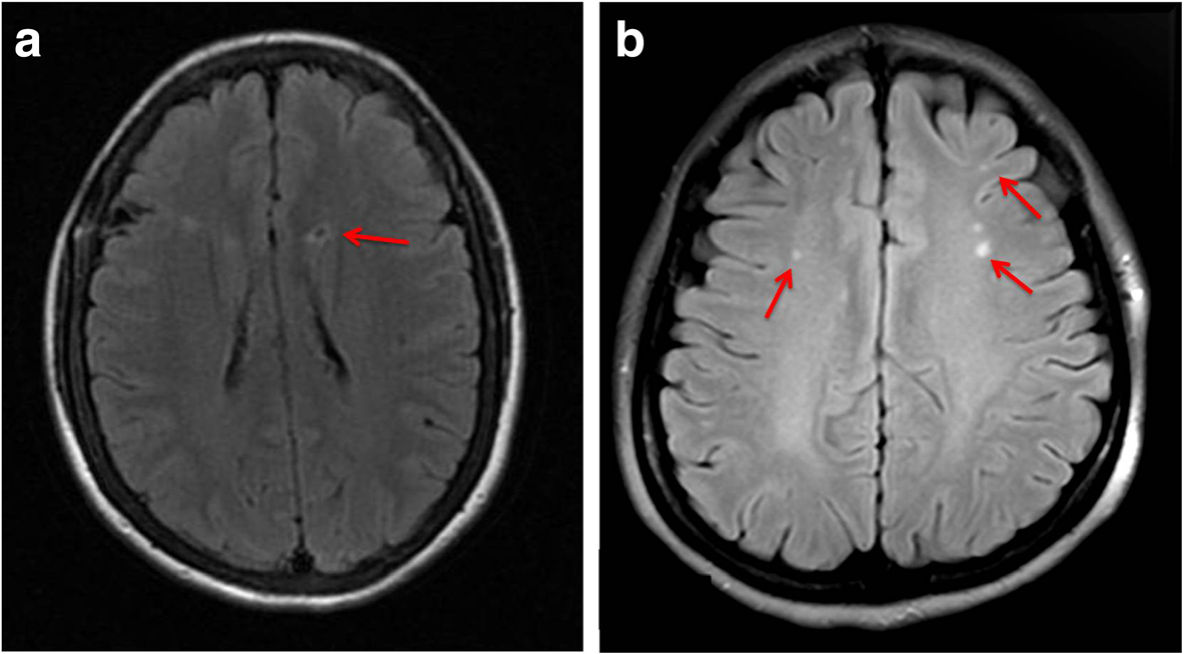
**a** A subclinical brain infarction in anterior circulation. **b** The white matter hyperintensity in deep white matter

We used 1.5-T scanners for whole-brain MRI, consisting of T1-weighted images (T1WI), T2-weighted images (T2WI), and fluid attenuated inversion recovery (FLAIR) images. SBIs were defined as non-mass parenchymal defects with a vascular distribution, isointense to cerebrospinal fluid signal on all sequences, and when supratentorial, surrounded by a hyperintense rim on FLAIR images, as shown in Fig. 2a. In the basal ganglia, only parenchymal defects larger than 3 mm in diameter were considered in order to exclude nonspecific lesions [5]. The SBI should be distinct from dilated vascular space (Virchow−Robin space) based on location, shape, size, and absence of a hyperintense border [6]. The location and number of the infarctions were recorded.

WMHs were defined as clearly hyperintense areas relative to surrounding white matter on both FLAIR and T2WI and were identified by simultaneous inspection of both aligned images (Fig. 2b) [18]. T2 hyperintense lesions that were not hyperintense on FLAIR images were considered as enlarged Virchow–Robin spaces. Periventricular WMHs (pvWMHs) were assessed in three regions (frontal and posterior horns and bands); dWMHs were located in deep white-matter tracts and were not attached to lateral ventricle lesions [5, 6].

Using the above-mentioned criteria, all of the neuroimages from a multicentre database were read by two trained neurologists, who were blinded to any clinical data.

### Statistical analysis

We used Pearson’s χ2 test to analyse discontinuous variables. For analysis of continuous variables, unpaired *t*-tests were used. The *p*-values were two-tailed, and *p* ≤ 0.05 was considered statistical significant. We used binary logistic regression models (odds ratio [OR], 95% confidence interval [CI]) to control age, sex, the main risk factors of vascular disease and aura for MRI outcomes among different groups, which were divided by the grade or type of RLS. All analyses were performed using IBM SPSS Statistics 20.0.

## Results

**Fig. 3:**
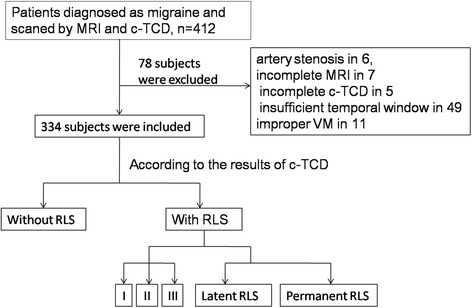
Flow chart of patient enrolment

This study included 412 migraineurs; 78 subjects were excluded (due to artery stenosis in 6, incomplete MRI in 7, incomplete c-TCD in 5, insufficient temporal window in 49, and improper VM execution in 11) (Fig. 3). Among the 334 subjects enrolled in our study, 224 subjects had a RLS (mean age 42.49 ± 10.97 years; 160 females) and 110 had no RLS (mean age 44.03 ± 10.12 years, 81 females). Demographic and clinical data of the subjects in groups with and without RLS are summarized in Table 1. Except that the prevalence of aura was higher in patients with RLS than in those without RLS (29.9% vs. 17.3%, *p* = 0.013), there were no significant differences between the two groups.

**Table 1:** Characteristics of the patients

Characteristics	RLS	No RLS	*P* value
	(*n* = 224)	(*n* = 110)	
Demographics			
Age (mean ± SD, years)	42.49 ± 10.97	44.03 ± 10.12	0.147^♯^
Female	160 (71.4)	81 (73.6)	0.672^*^
The main risk factors of vascular disease			
BMI (mean ± SD)	22.98 ± 3.32	20.03 ± 3.61	0.281^♯^
Hypertension	25 (11.2)	15 (13.6)	0.513^*^
Diabetes	5 (2.2)	5 (4.5)	0.244^*^
Heart disease	6 (2.7)	4 (3.6)	0.629^*^
Smoking			0.627^*^
Never	189 (84.4)	97 (88.2)	
Current	23 (10.3)	8 (7.3)	
Former	12 (5.4)	5 (4.5)	
Headache characteristics			
Aura	67 (29.9)	19 (17.3)	0.013^*^
Family history of headache	65 (29.0)	26 (23.6)	0.299^*^
Age of onset	31.01 ± 11.85	32.65 ± 11.92	0.776^♯^
Years from the first attack	11.84 ± 11.16	11.39 ± 10.45	0.731^♯^
MRI Findings			
			0.265^*^
SBI	20 (8.9)	6 (5.5)	
			0.066^※^
Posterior circulation only	10 (4.5)	1 (0.9)	0.166^*^
Single silent brain infarct	12 (5.4)	4 (3.6)	
			0.355^*^
WMHs	122 (54.5)	54 (49.1)	
			0.543^※^
pvWMHs	7 (3.1)	2 (1.8)	
dWMHs	94 (42.0)	46 (41.8)	0.980^*^
dWMHs and pvWMHs	21 (9.4)	6 (5.5)	

*Abbreviations*: *BMI* body mass index, *MRI* magnetic resonance imaging, *SBI* silent brain infarctions, *WMHs* white matter hyperintensities, *pvWMHs* periventricular WMHs, *dWMHs* deep WMHs

Data are presented as n (%) unless otherwise specified

^*^Pearson’s χ2, unadjusted for aura;

^♯^unpaired t-test

^※^P for trend computed by binary logistic regression

We found 26 subjects with SBI among the migraineurs in this study; in 61.5% (16/26), the SBI was a single lesion. Compared with the no-RLS group, SBI (8.9% vs. 5.5%, *p* = 0.265) and WMHs (54.5% vs. 49.1%, *p* = 0.355) did not increase with RLS in migraineurs (Table 1). These results were still not statistically significant after adjustment for aura.

When comparing grade by grade, we found a p for trend for SBI = 0.066 (OR = 1.43; 95%CI: 0.98−2.09) and a p for trend for WMHs = 0.543, (OR = 1.07; 95%CI:0.86−1.32). The incidence of SBI in the posterior circulation of migraineurs with RLS was not different from that in those without RLS (*p* = 0.166). The presence of dWMHs did not increase with the appearance of RLS (42.0% vs. 41.8%, *p* = 0.980).

However, there was a statistically significant difference in the prevalence of SBI when the subjects were divided by Grade (0−III) (Table 2). The presence of SBI in the Grade III group (curtain RLS) was higher than that in the Grade 0 group (16.7% vs. 5.5%, *p* = 0.032, OR = 3.47; 95%CI: 1.12−10.76), when controlling for age, sex, aura, and the main vascular disease risk factors in binary logistic regression analysis (Additional file 1). We identified curtain RLS (Grade III) as a risk factor for SBI in migraineurs.

**Table 2:** Prevalence of MRI findings by size of RLS

	SBI		WMHs	
	*n* (%)	OR(95%CI)	*n* (%)	OR(95%CI)
Grade 0 *n* = 110	6 (5.5)	–	54 (49.1)	–
Grade I *n* = 95	7 (7.4)	1.60 (0.48−5.31) *P* = 0.447	53 (55.8)	1.63(0.89−3.00) *P* = 0.114
GradeII *n* = 63	2 (3.2)	0.38(0.07−2.20) *P* = 0.281	34 (54.0)	1.28 (0.63−2.57) *P* = 0.497
Grade III *n* = 66	11 (16.7)	3.47(1.12−10.76) *P* = 0.032	35 (53.0)	1.22 (0.62−2.41) *P* = 0.563

*Abbreviations*: *MRI* magnetic resonance imaging, *RLS* right-to-left shunt, *SBI* silent brain infarctions, *WMHs* white matter hyperintensities

Odds ratio (OR) is computed by binary logistic regression, as compared to grade 0

When comparing permanent RLS and latent RLS with the group without RLS (Table 3), the incidence of SBI and WMHs were not statistically significantly different.

**Table 3:** Prevalence of MRI findings by subtype of RLS

	SBI		WMHs	
	*n* (%)	OR(95%CI)	*n* (%)	OR(95%CI)
No RLS *n* = 110	6 (5.5)	–	54 (49.1)	–
Latent RLS *n* = 106	6 (4.7)	2.53(0.87−7.40) *P* = 0.090	60 (56.6)	1.26(0.70−2.24) *P* = 0.440
Permanent RLS *n* = 118	14 (11.9)	0.89(0.25−3.14) *P* = 0.851	62 (52.5)	1.58(0.88−2.84) *P* = 0.129

*Abbreviations*: *MRI* magnetic resonance imaging, *RLS* right-to-left shunt, *SBI* silent brain infarctions, *WMHs* white matter hyperintensities

OR is computed by binary logistic regression compared to no RLS

## Discussion

In our study, SBI or WMHs did not increase with the size of RLS, but when the RLS was presented as a curtain pattern, it appeared to be a risk factor for SBI in migraineurs in our study. There was no association between the presence of a RLS and the presence of WMHs in migraineurs. The presence of RLS per se did not increase either SBI or posterior cerebral circulation in migraineurs. The subtype of RLS (permanent and latent) had no effect on SBI or WMHs.

### Curtain RLS and SBI in migraineurs

In order to determine the underlying relationship between the size of the RLS and SBI, we classified RLS into four grades according to the number of MBs. In total, of the 19.8% (66/334) individuals with curtain RLS, 16.7% (11/66) showed SBI. In the group without RLS, SBI was found in 5.5% (6/110) of the subjects. Compared to the no-RLS group, the incidence of SBI in the curtain pattern group was 2.4 times higher.

The source of the infarctions in patients with migraine is uncertain. There are several mechanisms underlying migraine-stroke, such as cortical spreading depression (CSD) [19], shared genetic risk factors for migraine and stroke, vasoconstrictor medications taken to treat headache [20], repeated or prolonged reduction of perfusion pressure, and reduced blood flow [21],which may produce thrombosis, embolism, or ischemic events. RLS, such as PFO, was closely associated with migraine [9, 15], and was also likely to increase ischemic brain lesions through paradoxical embolism, potentially leading to migraine attack or stroke. A case−control analysis denied the role of PFO in migraine-associated silent infarctions and ischemic stroke [22]. However, due to the relatively small sample size, they did not perform subgroup analysis based on the size of the PFO. In 1998, Serena et al. conducted a case−control study in which the prevalence of RLS in a nonselected group of patients consecutively admitted for cerebral infarction and TIA was compared with that in healthy control subjects, in order to study the relationship between the magnitude of RLS and stroke subtypes. Surprisingly they found that the curtain pattern was detected only in those with CS, but found no significant association between small RLS and the risk of stroke [16].

This study emphasized the importance of paying attention to curtain RLS. In a previous study about RLS subtypes in Chinese patients with CS, the results suggested that a large RLS was a pathological condition in CS [13]. This is not unexpected if we assume that a larger RLS would increase the risk of paradoxical embolism. Moreover, it has been found that dynamic cerebral autoregulation is impaired in migraineurs with a large RLS, and this may represent a potential mechanism linking RLS, migraine, and CS [23].

### Permanent RLS and SBI in migraineurs

Permanent RLS, which was regarded as dangerous RLS, was reported to increase ischemic cerebral lesions and previous recurrent stroke [24].Given that permanent RLS could occur during rest and without the induction of VM or similar actions, we could hypothesize that permanent RLS may pose a higher risk of paradoxical embolism. In our study, the frequency of SBI was in fact higher in the permanent RLS group than in the latent RLS and no-RLS group (11.9% vs. 4.7% vs. 5.5%), although the differences were not statistically significant. A larger sample should be established to confirm the role of permanent RLS in the occurrence of SBI.

### RLS and SBI located in the posterior circulation in migraineurs

Kruit et al. found that the prevalence of SBI located in the posterior circulation was higher in migraineurs, particularly when located in the cerebellum, in a population-based MRI CAMERA study [25]. However, RLS is reportedly not associated with specific ischemic patterns [26]. Our study did not find a higher frequency of SBI in the posterior circulation in the RLS group than in the no-RLS group. Due to the limited number of samples with SBI, we did not investigate the relationship between different RLS sizes and SBI in the posterior circulation further, and therefore, we cannot exclude an underlying connection.

### RLS and WMHs in migraineurs

It has been reported that migraine patients have a 4-fold higher risk of having white matter lesions than no-migraine subjects [27], and dWMHs have attracted much attention [1]. However, the mechanisms underlying white matter lesions are not completely understood and they might be associated with ischemic complications of various microvascular processes, such as ischemia, oxidative stress, energy deprivation, hypoglycaemia, or platelet hypercoagulability [6].In terms of RLS and WMHs, Del Sette et al. have reported that RLS does not increase the number and volume of WMHs in migraineurs [28]. In our study, we only focused on the presence and location of WMHs and did not measure the number and the load of lesions. We found no relationship between RLS and the presence of WMHs or dWMHs in migraineurs, which is in line with the findings of other studies [5]. However, a study in South Korea found that small dWMHs were associated with RLS in migraineurs [29]. It might be inferred that a stronger relationship is likely to exist between WMHs and RLS.

### The effect of RLS closure

The effect of RLS closure as a treatment has not been clear. RLS closure may reduce stroke recurrence and migraine attacks [30–32]. However, numerous randomized clinical trials have reported that, as a secondary prevention of cryptogenic embolism, PFO closure did not significantly reduce the risk of recurrent embolic events or death, as compared with medical therapy [33, 34]. In a study published in the New England Journal of Medicine in 2013 [33], the primary end-point, such as death, nonfatal stroke, TIA, or peripheral embolism, occurred at a frequency of 3.4% (7/204) in the PFO-closure group; this low-probability event requires validation in a sizeable sample. Given the results of our study, it could be considered whether the selection of patients with curtain RLS for PFO-closure treatment may improve the preventative effect. Randomized controlled trials may clarify this, and may better reflect the role of curtain RLS in mediating migraine and silent brain lesions.

### Strengths and limitations

In the past, in similar studies about the relationship between RLS and subclinical cerebral lesions, investigators generally classified the size of RLS as absent, small, medium, or large, according to the number of MBs observed. However, we singled out curtain RLS alone and identified it as a risk factor for SBI. In addition, a strength of our study is its unified multicentre design. We included well-defined migraine patients with or without aura, and excluded individuals with other types of headaches, e.g., chronic daily headache, tension headache, or cluster headache. We considered the subjects’ demographics, medical history, and migraine status by means of a questionnaire, and the complete c-TCD and MRI evaluation data of all patients.

Yet, our study also had some potential limitations. Overall, there were only 26 patients with SBI; the incidence of SBI in subjects with RLS was only 8.9%, which was a low-probability event and might result in inadequate statistical power. The number of subjects with the different grades of RLS was not sufficiently large, and the sample size of our study (*n* = 334) was not quite adequate to draw concrete conclusions. Secondly, c-TCD tests and MRI scans for subjects were performed in different centres, which led to unavoidable differences in sensitivity. In addition, the data were obtained in patients from hospital departments, who may have been more severely affected than the average migraineur. Thus, it is uncertain whether and to what degree these conclusions can be applied to all migraineurs. Moreover, our study was based on real-world observations, and thus we cannot discriminate the real causes of embolism. Further studies are needed to expand the sample size to verify that curtain RLS is indeed a risk factor for SBI in migraine. Furthermore, it should be investigated whether the prevalence of SBI in migraineurs with curtain RLS is associated with aura, and whether curtain RLS closure has a protective effect.

## Conclusions

In the present study, we concluded that subclinical ischemic lesions were neither more prevalent in migraineurs with RLS than in migraineurs without RLS, nor increased with the size of the RLS. Nevertheless, curtain RLS may be a risk factor for silent brain infarctions.

## Additional file

Additional file 1: Binary logistic regression test for possible factors of SBI. (DOCX 16 kb)

## Abbreviations

BMI: Body mass index
CI: Confidence interval
CS: Cryptogenic stroke
c-TCD: contrast-enhanced TCD
dWMHs: deep WMHs
FLAIR: Fluid attenuated inversion recovery
MA: Migraine with aura
MBs: Microbubbles
MCA: Middle cerebral artery
MRI: Magnetic resonance imaging
OR: Odds ratio
PFO: Patent foramen ovale
pvWMHs: periventricular WMHs
RLS: Right-to-left shunt
SBI: Silent brain infarctions
T1WI: T1-weighted image
T2WI: T2-weighted image
TCD: Transcranial Doppler
VM: Valsalva manoeuvre
WMHs: White matter hyperintensities
